# *N*-[4-(Dimethyl­amino)­benzyl­idene]-3,4-dimethyl­isoxazol-5-amine

**DOI:** 10.1107/S1600536810023780

**Published:** 2010-06-26

**Authors:** Abdullah M. Asiri, Salman A. Khan, Kong Wai Tan, Seik Weng Ng

**Affiliations:** a Chemistry Department, Faculty of Science, King Abdul Aziz University, PO Box 80203, Jeddah 21589, Saudi Arabia; b Department of Chemistry, University of Malaya, 50603 Kuala Lumpur, Malaysia

## Abstract

The aromatic rings attached to the azomethine double bond in the title compound, C_14_H_17_N_3_O, are *trans* to each other [C—C=N—C torsion angle = 179.5 (1)°], and they are approximately coplanar [dihedral angle between the five- and six-membered rings = 13.7 (1)°].

## Related literature

For the spectroscopic characterization of a related Schiff base, see: Asiri *et al.* (2010 ▶).
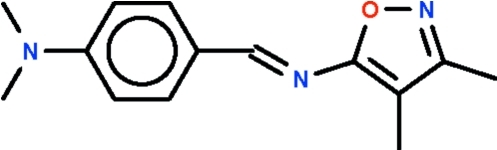


## Experimental

### 

#### Crystal data

C_14_H_17_N_3_O
*M* *_r_* = 243.31
Triclinic, 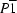

*a* = 6.5772 (6) Å
*b* = 9.1246 (9) Å
*c* = 10.538 (1) Å
α = 92.995 (1)°
β = 95.183 (1)°
γ = 90.873 (1)°
*V* = 628.86 (10) Å^3^
*Z* = 2
Mo *K*α radiation
μ = 0.08 mm^−1^
*T* = 100 K
0.35 × 0.15 × 0.10 mm

#### Data collection

Bruker SMART APEX diffractometer
6092 measured reflections
2866 independent reflections
2401 reflections with *I* > 2σ(*I*)
*R*_int_ = 0.023

#### Refinement

*R*[*F* ^2^ > 2σ(*F* ^2^)] = 0.039
*wR*(*F* ^2^) = 0.113
*S* = 1.04
2866 reflections
168 parameters
H-atom parameters constrained
Δρ_max_ = 0.25 e Å^−3^
Δρ_min_ = −0.24 e Å^−3^

### 

Data collection: *APEX2* (Bruker, 2009 ▶); cell refinement: *SAINT* (Bruker, 2009 ▶); data reduction: *SAINT*; program(s) used to solve structure: *SHELXS97* (Sheldrick, 2008 ▶); program(s) used to refine structure: *SHELXL97* (Sheldrick, 2008 ▶); molecular graphics: *X-SEED* (Barbour, 2001 ▶); software used to prepare material for publication: *publCIF* (Westrip, 2010 ▶).

## Supplementary Material

Crystal structure: contains datablocks global, I. DOI: 10.1107/S1600536810023780/jh2170sup1.cif

Structure factors: contains datablocks I. DOI: 10.1107/S1600536810023780/jh2170Isup2.hkl

Additional supplementary materials:  crystallographic information; 3D view; checkCIF report

## Figures and Tables

**Fig. 1.**
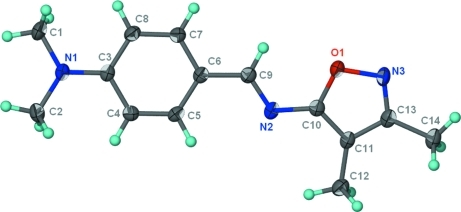
Thermal ellipsoid plot (Barbour, 2001) of C14H13N3O at the 70% probability level; hydrogen atoms are drawn as spheres of arbitrary radius.

## supplementary crystallographic information

### Comment

Although there is a large number of crystal structure studies of Schiff bases derived by condensing an aromatic aldehyde and an aromatic amine, there has not been any structural report on the condensation product involving 5-amino-3,4-dimethylisoxazole, a commerically available chemical. We have recently reported the spectroscopic characterization of the *N*-ethylcarbazole-3-aldehyde condensation product of this amine (Asiri *et al.*, 2010). The 4-dimethylaminobenzaldehyde condensation product (Scheme I, Fig. 1) features an azomethine double-bond whose aromatic substituents are located in *trans* positions. The rings are coplanar [C–C═N–C torsion angle 179.5 (1) °].

### Experimental

5-Amino-3,4-dimethylisoxazole (0.36 g, 3.2 mol) and *N*,*N*-dimethylaminobenzaldehyde (0.5 g, 3.2 mol) were heated in methanol (15 ml) for 5 h. The solvent was removed and the solid material recrystallized from methanol to give the crystalline Schiff base.

### Refinement

Carbon-bound H-atoms were placed in calculated positions [C–H 0.95 to 0.98 Å, *U*(H) 1.2 to 1.5*U*_eq_(C)] and were included in the refinement in the riding model approximation.

### Crystal data

**Table:**

C_14_H_17_N_3_O	*Z* = 2
*M**_r_* = 243.31	*F*(000) = 260
Triclinic, *P*1	*D*_x_ = 1.285 Mg m^−^^3^
Hall symbol: -P 1	Mo *K*α radiation, λ = 0.71073 Å
*a* = 6.5772 (6) Å	Cell parameters from 2610 reflections
*b* = 9.1246 (9) Å	θ = 2.2–28.3°
*c* = 10.538 (1) Å	µ = 0.08 mm^−^^1^
α = 92.995 (1)°	*T* = 100 K
β = 95.183 (1)°	Prism, yellow
γ = 90.873 (1)°	0.35 × 0.15 × 0.10 mm
*V* = 628.86 (10) Å^3^	

### Data collection

**Table:**

Bruker SMART APEX diffractometer	2401 reflections with *I* > 2σ(*I*)
Radiation source: fine-focus sealed tube	*R*_int_ = 0.023
graphite	θ_max_ = 27.5°, θ_min_ = 1.9°
ω scans	*h* = −8→8
6092 measured reflections	*k* = −11→11
2866 independent reflections	*l* = −13→13

### Refinement

**Table:**

Refinement on *F*^2^	Secondary atom site location: difference Fourier map
Least-squares matrix: full	Hydrogen site location: inferred from neighbouring sites
*R*[*F*^2^ > 2σ(*F*^2^)] = 0.039	H-atom parameters constrained
*wR*(*F*^2^) = 0.113	*w* = 1/[σ^2^(*F*_o_^2^) + (0.0652*P*)^2^ + 0.0819*P*] where *P* = (*F*_o_^2^ + 2*F*_c_^2^)/3
*S* = 1.04	(Δ/σ)_max_ = 0.001
2866 reflections	Δρ_max_ = 0.25 e Å^−^^3^
168 parameters	Δρ_min_ = −0.24 e Å^−^^3^
0 restraints	Extinction correction: *SHELXL97* (Sheldrick, 2008), Fc^*^=kFc[1+0.001xFc^2^λ^3^/sin(2θ)]^-1/4^
Primary atom site location: structure-invariant direct methods	Extinction coefficient: 0.024 (5)

### Fractional atomic coordinates and isotropic or equivalent isotropic displacement parameters (Å^2^)

**Table:**

	*x*	*y*	*z*	*U*_iso_*/*U*_eq_	
O1	0.34307 (12)	0.64426 (8)	0.20359 (7)	0.0212 (2)	
N2	0.46514 (14)	0.43648 (10)	0.31374 (9)	0.0188 (2)	
N3	0.41200 (15)	0.73832 (10)	0.11248 (9)	0.0229 (2)	
N1	0.13966 (15)	0.02064 (10)	0.73244 (9)	0.0214 (2)	
C1	−0.06808 (18)	−0.00795 (14)	0.76262 (12)	0.0275 (3)	
H1A	−0.1258	0.0831	0.7959	0.041*	
H1B	−0.0673	−0.0816	0.8271	0.041*	
H1C	−0.1511	−0.0446	0.6852	0.041*	
C3	0.17610 (17)	0.11519 (11)	0.64040 (10)	0.0175 (2)	
C2	0.29986 (19)	−0.07284 (12)	0.78465 (12)	0.0251 (3)	
H2A	0.3270	−0.1499	0.7204	0.038*	
H2B	0.2561	−0.1179	0.8604	0.038*	
H2C	0.4245	−0.0138	0.8084	0.038*	
C4	0.37269 (17)	0.13022 (12)	0.59705 (10)	0.0189 (2)	
H4	0.4797	0.0699	0.6293	0.023*	
C5	0.41094 (17)	0.23055 (12)	0.50920 (10)	0.0184 (2)	
H5	0.5447	0.2391	0.4827	0.022*	
C6	0.25736 (16)	0.32079 (12)	0.45762 (10)	0.0176 (2)	
C7	0.06149 (17)	0.30343 (12)	0.49799 (11)	0.0194 (2)	
H7	−0.0457	0.3622	0.4634	0.023*	
C8	0.01988 (17)	0.20356 (12)	0.58643 (11)	0.0201 (2)	
H8	−0.1148	0.1941	0.6113	0.024*	
C9	0.29510 (17)	0.42739 (12)	0.36576 (10)	0.0185 (2)	
H9	0.1903	0.4939	0.3424	0.022*	
C10	0.49005 (17)	0.54187 (12)	0.22757 (10)	0.0180 (2)	
C11	0.64745 (16)	0.56405 (11)	0.15557 (10)	0.0180 (2)	
C12	0.83736 (17)	0.47749 (13)	0.14888 (11)	0.0237 (3)	
H12A	0.8486	0.4097	0.2182	0.036*	
H12B	0.9564	0.5444	0.1574	0.036*	
H12C	0.8318	0.4215	0.0667	0.036*	
C13	0.58994 (17)	0.68813 (12)	0.08652 (10)	0.0197 (2)	
C14	0.71023 (19)	0.76199 (13)	−0.00676 (11)	0.0245 (3)	
H14A	0.7154	0.6972	−0.0835	0.037*	
H14B	0.8493	0.7833	0.0320	0.037*	
H14C	0.6448	0.8538	−0.0299	0.037*	

### Atomic displacement parameters (Å^2^)

**Table:**

	*U*^11^	*U*^22^	*U*^33^	*U*^12^	*U*^13^	*U*^23^
O1	0.0190 (4)	0.0238 (4)	0.0218 (4)	−0.0006 (3)	0.0047 (3)	0.0072 (3)
N2	0.0200 (5)	0.0196 (5)	0.0168 (5)	−0.0031 (4)	0.0033 (4)	0.0010 (3)
N3	0.0236 (5)	0.0256 (5)	0.0203 (5)	−0.0034 (4)	0.0033 (4)	0.0088 (4)
N1	0.0200 (5)	0.0231 (5)	0.0224 (5)	−0.0003 (4)	0.0047 (4)	0.0078 (4)
C1	0.0240 (6)	0.0296 (6)	0.0311 (7)	−0.0021 (5)	0.0093 (5)	0.0117 (5)
C3	0.0197 (6)	0.0169 (5)	0.0160 (5)	−0.0017 (4)	0.0026 (4)	0.0006 (4)
C2	0.0285 (6)	0.0216 (6)	0.0267 (6)	0.0039 (5)	0.0053 (5)	0.0092 (5)
C4	0.0176 (5)	0.0198 (5)	0.0193 (5)	0.0015 (4)	0.0022 (4)	0.0015 (4)
C5	0.0157 (5)	0.0208 (5)	0.0190 (5)	−0.0020 (4)	0.0039 (4)	−0.0001 (4)
C6	0.0179 (5)	0.0188 (5)	0.0159 (5)	−0.0020 (4)	0.0018 (4)	0.0001 (4)
C7	0.0165 (5)	0.0224 (5)	0.0197 (5)	0.0010 (4)	0.0015 (4)	0.0034 (4)
C8	0.0160 (5)	0.0241 (6)	0.0207 (6)	−0.0013 (4)	0.0038 (4)	0.0026 (4)
C9	0.0173 (5)	0.0211 (5)	0.0168 (5)	−0.0012 (4)	0.0007 (4)	0.0012 (4)
C10	0.0182 (5)	0.0189 (5)	0.0165 (5)	−0.0020 (4)	0.0000 (4)	0.0003 (4)
C11	0.0184 (5)	0.0206 (5)	0.0146 (5)	−0.0047 (4)	0.0010 (4)	0.0004 (4)
C12	0.0197 (6)	0.0280 (6)	0.0242 (6)	−0.0016 (4)	0.0045 (5)	0.0038 (5)
C13	0.0205 (6)	0.0227 (5)	0.0156 (5)	−0.0052 (4)	0.0003 (4)	0.0006 (4)
C14	0.0267 (6)	0.0273 (6)	0.0201 (6)	−0.0054 (5)	0.0039 (5)	0.0046 (5)

### Geometric parameters (Å, °)

**Table:**

O1—C10	1.3709 (13)	C5—C6	1.4025 (15)
O1—N3	1.4201 (11)	C5—H5	0.9500
N2—C9	1.2926 (14)	C6—C7	1.4020 (15)
N2—C10	1.3745 (14)	C6—C9	1.4419 (15)
N3—C13	1.3097 (15)	C7—C8	1.3788 (15)
N1—C3	1.3659 (14)	C7—H7	0.9500
N1—C2	1.4533 (14)	C8—H8	0.9500
N1—C1	1.4534 (14)	C9—H9	0.9500
C1—H1A	0.9800	C10—C11	1.3565 (15)
C1—H1B	0.9800	C11—C13	1.4155 (15)
C1—H1C	0.9800	C11—C12	1.4930 (15)
C3—C8	1.4132 (15)	C12—H12A	0.9800
C3—C4	1.4168 (15)	C12—H12B	0.9800
C2—H2A	0.9800	C12—H12C	0.9800
C2—H2B	0.9800	C13—C14	1.4966 (15)
C2—H2C	0.9800	C14—H14A	0.9800
C4—C5	1.3718 (15)	C14—H14B	0.9800
C4—H4	0.9500	C14—H14C	0.9800
			
C10—O1—N3	107.86 (8)	C8—C7—C6	121.91 (10)
C9—N2—C10	119.52 (10)	C8—C7—H7	119.0
C13—N3—O1	105.28 (9)	C6—C7—H7	119.0
C3—N1—C2	120.76 (9)	C7—C8—C3	120.48 (10)
C3—N1—C1	120.12 (9)	C7—C8—H8	119.8
C2—N1—C1	118.15 (9)	C3—C8—H8	119.8
N1—C1—H1A	109.5	N2—C9—C6	122.97 (10)
N1—C1—H1B	109.5	N2—C9—H9	118.5
H1A—C1—H1B	109.5	C6—C9—H9	118.5
N1—C1—H1C	109.5	C11—C10—O1	109.95 (10)
H1A—C1—H1C	109.5	C11—C10—N2	129.32 (10)
H1B—C1—H1C	109.5	O1—C10—N2	120.73 (9)
N1—C3—C8	121.22 (10)	C10—C11—C13	104.10 (10)
N1—C3—C4	121.27 (10)	C10—C11—C12	128.36 (10)
C8—C3—C4	117.50 (10)	C13—C11—C12	127.54 (10)
N1—C2—H2A	109.5	C11—C12—H12A	109.5
N1—C2—H2B	109.5	C11—C12—H12B	109.5
H2A—C2—H2B	109.5	H12A—C12—H12B	109.5
N1—C2—H2C	109.5	C11—C12—H12C	109.5
H2A—C2—H2C	109.5	H12A—C12—H12C	109.5
H2B—C2—H2C	109.5	H12B—C12—H12C	109.5
C5—C4—C3	121.04 (10)	N3—C13—C11	112.81 (10)
C5—C4—H4	119.5	N3—C13—C14	120.31 (10)
C3—C4—H4	119.5	C11—C13—C14	126.88 (11)
C4—C5—C6	121.58 (10)	C13—C14—H14A	109.5
C4—C5—H5	119.2	C13—C14—H14B	109.5
C6—C5—H5	119.2	H14A—C14—H14B	109.5
C7—C6—C5	117.44 (10)	C13—C14—H14C	109.5
C7—C6—C9	120.23 (10)	H14A—C14—H14C	109.5
C5—C6—C9	122.33 (10)	H14B—C14—H14C	109.5
			
C10—O1—N3—C13	0.59 (11)	C7—C6—C9—N2	−171.57 (10)
C2—N1—C3—C8	178.20 (10)	C5—C6—C9—N2	7.97 (17)
C1—N1—C3—C8	9.66 (16)	N3—O1—C10—C11	−0.86 (11)
C2—N1—C3—C4	−2.91 (16)	N3—O1—C10—N2	179.38 (9)
C1—N1—C3—C4	−171.45 (10)	C9—N2—C10—C11	−174.71 (11)
N1—C3—C4—C5	−176.74 (10)	C9—N2—C10—O1	5.00 (15)
C8—C3—C4—C5	2.19 (16)	O1—C10—C11—C13	0.75 (12)
C3—C4—C5—C6	−0.80 (17)	N2—C10—C11—C13	−179.51 (10)
C4—C5—C6—C7	−0.81 (16)	O1—C10—C11—C12	−178.70 (10)
C4—C5—C6—C9	179.64 (9)	N2—C10—C11—C12	1.03 (19)
C5—C6—C7—C8	1.00 (16)	O1—N3—C13—C11	−0.13 (12)
C9—C6—C7—C8	−179.45 (10)	O1—N3—C13—C14	−179.61 (9)
C6—C7—C8—C3	0.44 (17)	C10—C11—C13—N3	−0.38 (13)
N1—C3—C8—C7	176.93 (10)	C12—C11—C13—N3	179.08 (10)
C4—C3—C8—C7	−2.01 (16)	C10—C11—C13—C14	179.06 (10)
C10—N2—C9—C6	−179.54 (9)	C12—C11—C13—C14	−1.47 (18)
